# Supplementary material for: Vibrio cholerae CsrA controls ToxR levels by increasing the stability and translation of toxR mRNA
Source: mBio. 2024 Nov 18;15(12):e02853-24. doi: 10.1128/mbio.02853-24 (PMC11633198; doi:10.1128/mbio.02853-24)
Supplement: Supplemental figures and tables — Tables S1–S3 and Figures S1–S5. [file mbio.02853-24-s0001.pdf]

Table S1. Bacterial strains and plasmids used in this study

Strain or plasmid	Description	Source or reference
Strains		
V. cholerae		
N16961	*V. cholerae* El Tor biotype	R. A. Finkelstein
N*csrA*.R6H	N16961 carrying *csrA*.R6H	(1)
N*toxR*.FS1	N16961 with a single T nucleotide insertion following the first in-frame ATG in *toxR*	This study
N*toxR*.FS2	N16961 with a single T nucleotide deletion following the first in-frame ATG in *toxR*	This study
N*toxR*.G1,2,3	N16961with mutations in *toxR* GGA motifs 1, 2, and 3	This study
N*toxR*.G3	N16961 with mutations in *toxR* GGA motif 3	This study
N*toxR*.G3,4	N16961 with mutations in *toxR* GGA motifs 3 and 4	This study
N*toxR*.G4,5	N16961with mutations in *toxR* GGA motifs 4 and 5	This study
N*toxR*.M1L	N16961 with mutation of first in-frame ATG to CTT in *toxR*	This study
N*toxR*.M13L	N16961 with mutation of second in-frame ATG to CTT in *toxR*	This study
N*toxR*.M1,13L	N16961 with mutation of both first and second in-frame ATGs to CTT in *toxR*	This study
N*toxR*.SDdown	N16961carrying mutations to weaken the predicted *toxR* Shine-Dalgarno (SD) sequence	This study
N*toxR*.SDup	N16961carrying mutations to strengthen the predicted *toxR* SD sequence	This study
N*toxR*.SDstem	N16961 carrying mutations to disrupt the putative *toxR* SD-sequestering stem-loop structure	This study
R6H.SDdown	N*csrA*.R6H carrying the *toxR* SDdown mutation	This study
R6H.SDup	N*csrA*.R6H carrying the *toxR* SDup mutation	This study
R6H.SDstem	N*csrA*.R6H carrying the *toxR* SDstem mutation	This study
SAC119	N16961 Δ*toxR*::*kan*	(2)
E. coli		
BL21 DE3	*E. coli* B-derived protein expression strain: F⁻ *ompT gal dcm lon* $hsdS_B(r_B^- m_B^-)$ λ(DE3)	(3)

Codon plus DE3 pRIL		Agilent Technologies
DH5α(λ*pir*)	cloning strain, host strain for pGP704 derivatives	J. Kaper

Plasmids

pCC1	Single-copy-no. cloning vector; Cam^r	Epicenter
pCVD442N	suicide vector pGP704 carrying *sacB*; Amp^r, Suc^s	(4)
pET16b	Bacterial expression vector	Novagen
pWKS30	Low-copy-no. cloning vector; Amp^r	(5)
pAMS33	pCVD442N carrying *toxR* with first in-frame ATG to CTT mutation	This study
pAMS34	pCVD442N carrying *toxR* with second in-frame ATG to CTT mutation	This study
pAMS35	pCVD442N carrying *toxR* with the FS1 frame shift mutation	This study
pAMS36	pCVD442N carrying *toxR* with the FS2 frame shift mutation	This study
pAMS37	pCVD442N carrying *toxR* with SDdown mutation	This study
pAMS38	pCVD442N carrying *toxR* with SDup mutation	This study
pAMS39	pCVD442N carrying *toxR* with the SDstem mutation	This study
pAMS41	pCVD442N carrying *toxR* with mutations in GGA motifs 1, 2, and 3	This study
pAMS42	pCVD442N carrying *toxR* with mutations in GGA motif 3	This study
pAMS43	pCVD442N carrying *toxR* with mutations in GGA motifs 3 and 4	This study
pAMS44	pCVD442N carrying *toxR* with mutations in GGA motifs 4 and 5	This study
pHTCsrA	pET16b encoding codon-optimized *V. cholerae* CsrA with an N-terminal 6xHis tag and a TEV cleavage site	This study
pFCsrA	pCC1 carrying *csrA*	This study
pRK793	TEV protease expression vector	(6)

Table S2. PCR primers used in this study

Name of primer	Primer Sequence (5'→3')[a]
htpG1	CCGCATCATCCATAATAAAGACGC
toxS2	CAGCAGAGAGAGCAGAAGAATACCC
toxR.ATG(1).F	GGGACATTAGCTTTTCGGATTAGG [M1L]
toxR.ATG(1).R	CCTAATCCGAAAAGCTAATGTCCC [M1L]
toxR.ATG(2).F	AAAAGAGATATCGCTTAGTCATATTGG [M13L]
toxR.ATG(2).R	CCAATATGACTAAGCGATATCTCTTTTGAG [M13L]
toxR.FS1.F	GGGACATTAGATGTTTCGGATTAGG
toxR.FS1.R	CCTAATCCGAAACATCTAATGTCCC
toxR.FS2.F	GGGACATTAGATGTCGGATTAGG
toxR.FS2.R	CCTAATCCGACATCTAATGTCCC
toxR.SDdown.F	ACACAACTCAAAACACATATCGATGAGTC
toxR.SDdown.R	CTCATCGATATGTGTTTTGAGTTGTGTCC
toxR.SDup.F	ACACAACTCAAAGGAGGTATCGATGAGTC
toxR.SDup.R	CTCATCGATACCTCCTTTGAGTTGTGTCC
toxR.SDstem.F	GGACACAAGTGAAAAGAGATATCGATGAG
toxR.SDstem.R	GATATCTCTTTTCACTTGTGTCCTAATCCG
G1,2,3mut.F	GTTTCAACATCTAGATACTTCAACATTAGATGTTCGGATTAGG
G1,2,3mut.R	TGTTGAAGTATCTAGATGTTGAAACACTCAGACTTTACTGG
G3mut.F	GACAGGGAGATACTTCAACATTAGATGTTCGG
G3mut.R	CCGAACATCTAATGTTGAAGTATCTCCCTGTCC
G3,4mut.F	GGGAGATACTTCAACATTAGATGTTCTCATTAGGACAC
G3,4mut.R	GTCCTAATGAGAACATCTAATGTTGAAGTATCTCCCTG
G4,5mut.F	GTTCTCATTATCACACAACTCAAAAGAG

G4,5mut.R	TGAGTTGTGTGATAATGAGAACATCTAATGTC
toxR-T7.F1	GCG AAT **TAA TAC GAC TCA CTA TAG G**GC ATT TTT ATC AAA GAA GAT AAA AAA ACC (the T7 promoter sequence is shown in bold)
toxR-T7.R1	**AAA CCC CTC CGT TTA GAG AGG GGT TAT GCT AG***T TA*C TCA CAC ACT TTG ATG GCA TCG (the T7 terminator sequence is shown in bold; the stop codon is italicized)

[a]Mutated nucleotides are underlined

Table S3. Biotinylated RNA oligonucleotides for RNA Electrophoretic Mobility Shift Assays (REMSAs)

Name of primer	Oligonucleotide Sequence (5'→3')[a]	Source and Reference
toxR 5'UTR	[Btn][b]GCAUUUUUAUCAAAGAAGAUAAAAAAACCAGUAAAGUCUGAGUGUUGGGACAGGGAGAUACUGGGACAUUAGAUGUUCGGAUUAGGACACAACUCAAAAGAGAUAUCGAUG	Ultramer®, IDT; this study
CsrB oligo	UUAGAUGCAGGGAGCACCUUUUAGUAGCUGGAAUGCUGCGA[Btn]	Sigma-Aldrich; this study
CsrB-mut	UUAGAUGCAUAUAGCACCUUUUAGUAGCUAUAAUGCUGCGA[Btn]	Sigma-Aldrich; this study
mut-left	UUAGAUGCAUAUAGCACCUUUUAGUAGCUGGAAUGCUGCGA[Btn]	Sigma-Aldrich; this study
mut-right	UUAGAUGCAGGGAGCACCUUUUAGUAGCUAUAAUGCUGCGA[Btn]	Sigma-Aldrich; this study
CsrB-left	UUAGAUGCAGGGAGCACCUUU[Btn]	Sigma-Aldrich; this study
CsrB-right	UUUUAGUAGCUGGAAUGCUGCGA[Btn]	Sigma-Aldrich; this study

[a]Mutated nucleotides are underlined
[b][Btn] denotes a biotinylation modification at the indicated primer end

SUPPLEMENTAL FIGURES

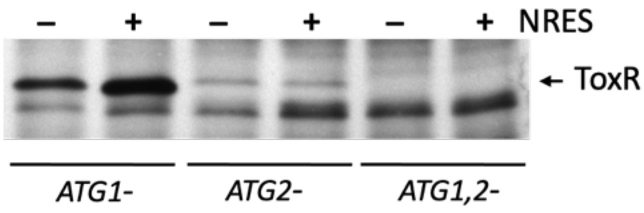

Figure S1. Overexposure of the anti-ToxR Western blot shown in Fig. 1B (top panel) shows the presence of a slower migrating ToxR band in the *ATG2-* single mutant, but not in the *ATG1,2-* double mutant. The wild-type strain N16961 (WT), the single mutants N*toxR*.M1L (*ATG1-*) and N*toxR*.M13L (*ATG2-*), and the double mutant N*toxR*.M1,13L (*ATG1,2-*) were grown in T medium with or without 12.5 mM NRES mix. Cells were harvested in mid-logarithmic phase, and whole cell preparations were resolved by SDS-PAGE and immunoblotted using polyclonal anti-ToxR antisera.

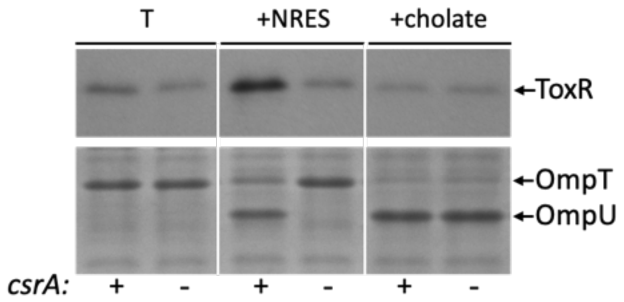

Figure S2. The bile acid cholate induces a CsrA-independent switch in porin production from OmpT to OmpU without increasing the level of the ToxR protein. The wild-type strain N16961 (*csrA+*) and the *csrA* mutant strain N*csrA*.R6H (*csrA-*) were grown in T medium with or without 12.5 mM NRES mix or 0.1% sodium cholate. Cells were harvested in mid-logarithmic phase, and whole cell preparations were resolved by SDS-PAGE and immunoblotted using polyclonal anti-ToxR antisera (top panel) or stained with Coomassie Blue to visualize the Omp proteins (bottom panel). The white vertical lines indicate that intervening lanes have been removed for clarity.

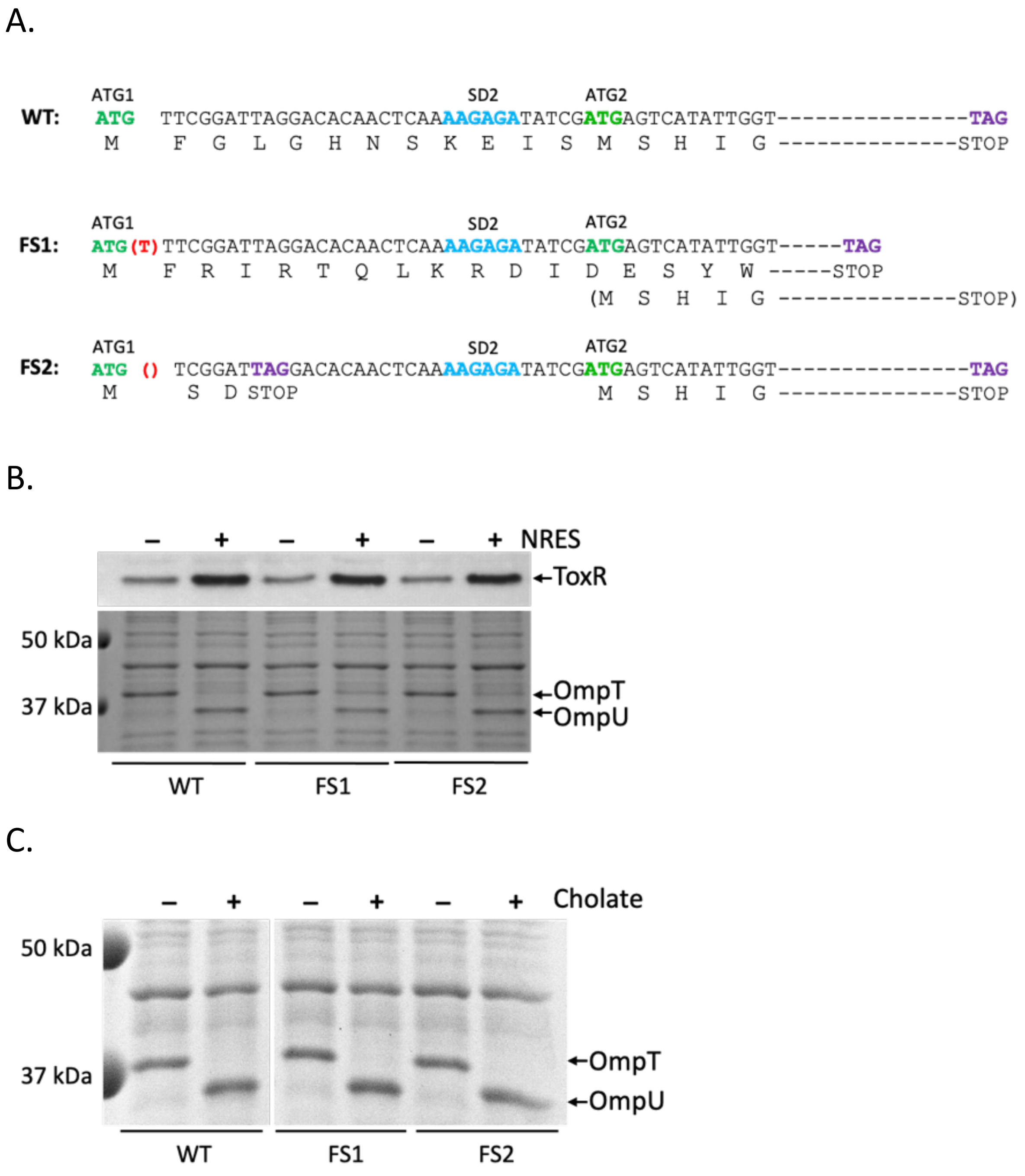

Figure S3. Evaluation of the open reading frame between the two in-frame ATG start codons in the *toxR* 5'UTR. (A) Schematic showing the nucleotide sequences and the encoded polypeptide sequences of the wild-type strain N16961 (WT) and the two frame-shift mutants, N*toxR*.FS1 (FS1)(added T shown in red parentheses) and N*toxR*.FS2 (FS2)(deleted T indicated by empty

red parentheses). A premature stop codon is indicated in purple in the FS2 sequence. (B) The strain N16961 (WT) and the frame-shift mutants N*toxR*.FS1 (FS1) and N*toxR*.FS2 (FS2) were grown to mid-logarithmic phase in T medium with or without 12.5 mM NRES mix. Whole cell preparations were resolved by SDS-PAGE and immunoblotted using polyclonal anti-ToxR antisera (top panel) or stained with Coomassie Blue to visualize the Omp proteins (bottom panel). (C) The wild-type strain N16961 (WT) and the frame-shift mutants N*toxR*.FS1 (FS1) and N*toxR*.FS2 (FS2) were grown to mid-logarithmic phase with or without sodium cholate, and whole cell preparations were resolved by SDS-PAGE and stained with Coomassie Blue to visualize the Omp proteins. The white vertical line indicates that intervening lanes have been removed for clarity.

A.

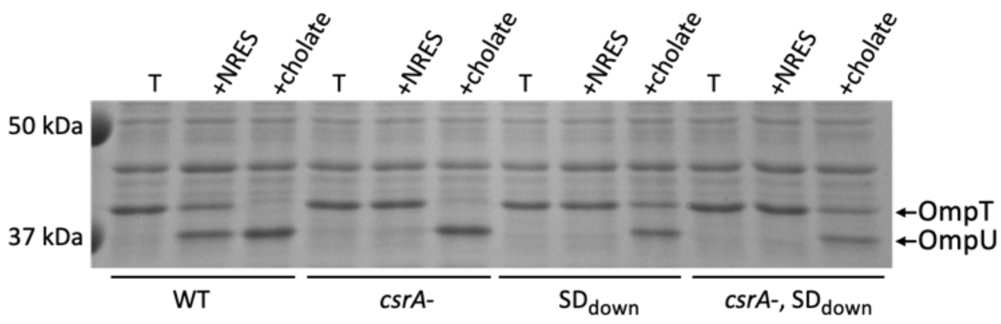
T
+NRES
+cholate
50 kDa
37 kDa
←OmpT
←OmpU
WT
csrA-
SDdown
csrA-, SDdown

B.

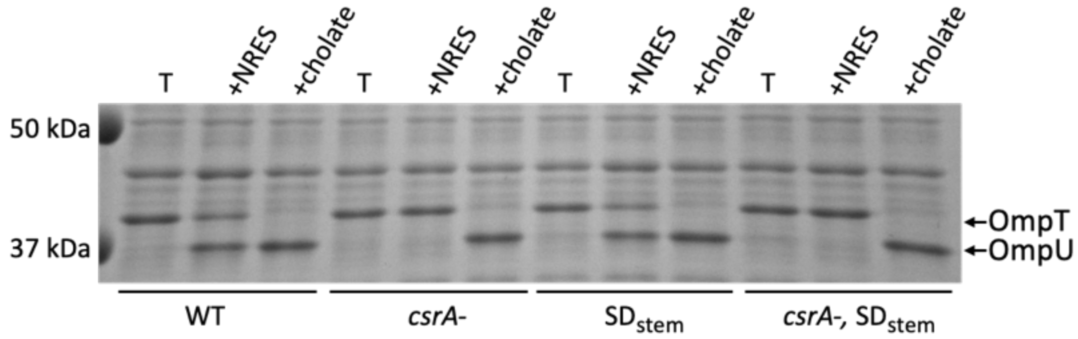
T
+NRES
+cholate
50 kDa
37 kDa
←OmpT
←OmpU
WT
csrA-
SDstem
csrA-, SDstem

C.

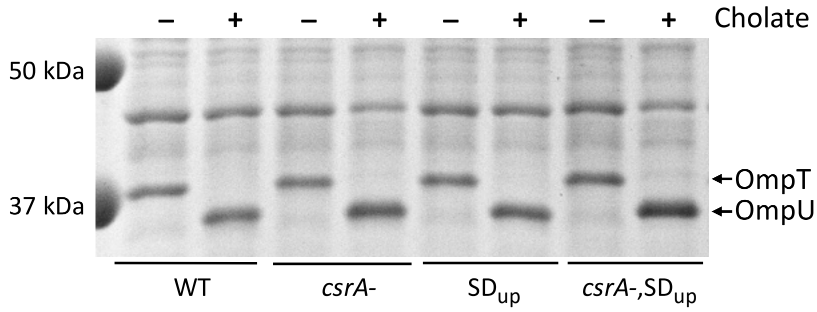
–
+
Cholate
50 kDa
37 kDa
←OmpT
←OmpU
WT
csrA-
SDup
csrA-,SDup

D.

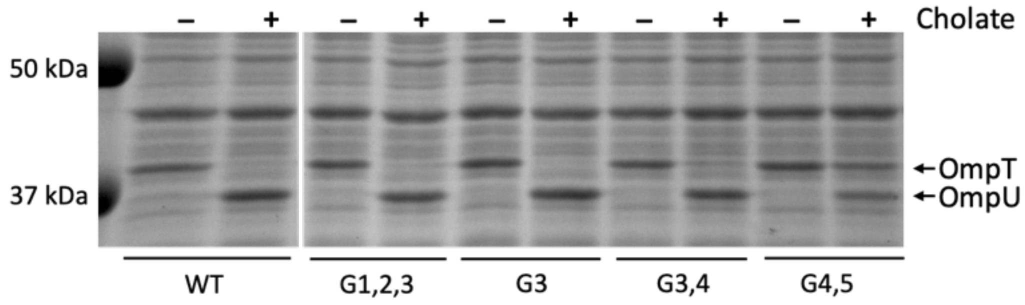

Figure S4. The effect of NRES and/or cholate on the Omp profiles of the *toxR* 5'UTR or SD sequence mutants in the wild-type or *csrA-* strain backgrounds. Strains were grown to mid-logarithmic phase in T medium with or without 12.5 mM NRES mix or 0.1% sodium cholate, as indicated. Whole cell preparations were resolved by SDS-PAGE and stained with Coomassie Blue to visualize the Omp proteins. (A-C) Strains shown are the wild-type strain N16961 (WT) and the *csrA* mutant strain N*csrA*.R6H (*csrA*-), together with (A): the N*toxR*.SDdown (SDdown) and R6H.SDdown (*csrA*-, SDdown) mutants; (B): the N*toxR*.SDstem (SDstem) and R6H.SDstem (*csrA*-, SDstem) mutants; or (C): the N*toxR*.SDup (SDup) and R6H.SDup (*csrA*-, SDup) mutants. (D) The wild-type strain (WT) and the GGA motif mutants N*toxR*.G1,2,3 (G1,2,3), N*toxR*.G3, (G3), N*toxR*.G3,4 (G3,4), and N*toxR*.G4,5 (G4,5) are shown. The vertical white line indicates that intervening lanes have been removed for clarity.

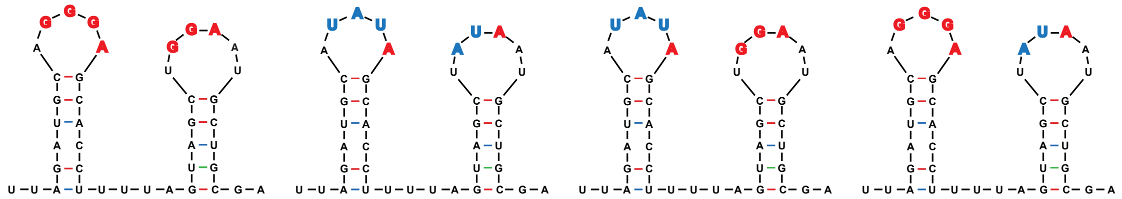

CsrB oligo CsrB-mut mut-left mut-right

Figure S5. Structures of in silico-folded CsrB oligos, showing two adjacent stem-loop structures with (G)GGA motifs located in the loops. The wild-type (G)GGA motif nucleotides are shown in red letters and the mutated (G)GGA motif nucleotides are shown in blue letters. The mutations did not alter the predicted folding of the CsrB oligos by mfold analysis (7).

REFERENCES

1. **Mey AR, Butz HA, Payne SM.** 2015. *Vibrio cholerae* CsrA Regulates ToxR Levels in Response to Amino Acids and Is Essential for Virulence. MBio **6:**e01064.
2. **Craig SA, Carpenter CD, Mey AR, Wyckoff EE, Payne SM.** 2011. Positive Regulation of the *Vibrio cholerae* Porin OmpT by Iron and Fur. J Bacteriol **193:**6505-6511.
3. **Studier FW, Moffatt BA.** 1986. Use of bacteriophage T7 RNA polymerase to direct selective high-level expression of cloned genes. J Mol Biol **189:**113-130.
4. **Wyckoff EE, Mey AR, Leimbach A, Fisher CF, Payne SM.** 2006. Characterization of ferric and ferrous iron transport systems in *Vibrio cholerae*. J Bacteriol **188:**6515-6523.
5. **Wang RF, Kushner SR.** 1991. Construction of versatile low-copy-number vectors for cloning, sequencing and gene expression in *Escherichia coli*. Gene **100:**195-199.
6. **Kapust RB, Tozser J, Fox JD, Anderson DE, Cherry S, Copeland TD, Waugh DS.** 2001. Tobacco etch virus protease: mechanism of autolysis and rational design of stable mutants with wild-type catalytic proficiency. Protein Eng **14:**993-1000.
7. **Zuker M.** 2003. Mfold web server for nucleic acid folding and hybridization prediction. Nucleic Acids Res **31:**3406-3415.
